# Draft Genome Sequence of Brevibacillus brevis LABIM17, a Biotechnologically Important Antimicrobial-Producing Bacterium

**DOI:** 10.1128/mra.00006-22

**Published:** 2022-02-22

**Authors:** Larissa de Medeiros Chagas, Gustavo Manoel Teixeira, Mirela Mosela, João Paulo de Oliveira, Maria Luiza Abreu Nicoletto, Rosiana Bertê, Daniel Vieira da Silva, Danilo Sipoli Sanches, Jacques Duílio Brancher, Ulisses Pereira Padua, Admilton Gonçalves de Oliveira

**Affiliations:** a Department of Microbiology, State University of Londrina, Londrina, Paraná, Brazil; b Federal University of Technology-Paraná, Cornélio Procópio, Paraná, Brazil; c Department of Computer Science, State University of Londrina, Londrina, Paraná, Brazil; d Department of Preventive Veterinary Medicine, State University of Londrina, Londrina, Paraná, Brazil; e Laboratory of Electron Microscopy and Microanalysis, State University of Londrina, Londrina, Paraná, Brazil; University of Arizona

## Abstract

Brevibacillus brevis LABIM17 is a bacterial isolate with biotechnological potential. Its draft genome sequence contains a chromosome of 5,950,202 bp, with 5,477 coding sequences, and exhibits 12 clusters involved in the production of secondary metabolites, which are likely responsible for its antimicrobial activity against several human and plant pathogens.

## ANNOUNCEMENT

Many microorganisms produce secondary metabolites, which represent a source for the development of biological products for plant disease control or new antibiotics for human disease control and/or other industrial applications. Throughout history, bioprospecting for biological agents has focused on traditional methods based on isolation and “blind” culture optimization. However, advances in next-generation sequencing technologies have offered a platform for the pan-genomic analysis of microorganisms down to the gene level (1). This strategy, in addition to classic methods, can accelerate the development of microbiological technologies. In anticipation of future in-depth genomic analyses and their potential applications, here we present genomic data on Brevibacillus brevis LABIM17, an antagonist of human and plant pathogens (2, 3).

For isolation purposes, a soil sample from a semideciduous secondary forest fragment in Londrina, Paraná, Brazil (23°19′37.8″S, 51°12′22.4″W), was dissolved (1:10) in saline solution (0.9%), shaken for 5 min, and heated at 80°C for 10 min. Serial dilutions of up to 10^−4^ were prepared, and 100-μL aliquots were spread on nutrient agar plates and incubated at 28°C for 48 h. A colony that exhibited natural antagonism was isolated, recorded (LABIM17), and deposited in the Microbial Culture Collection of the Laboratory of Microbial Biotechnology, State University of Londrina (Londrina, Paraná, Brazil). From the stock tube, LABIM17 was activated on a nutrient agar plate and incubated at 28°C for 48 h, and a single colony was selected for DNA isolation. The Quick-DNA miniprep kit (Zymo Research) was used for genomic DNA extraction, and the library was assembled using the Nextera XT DNA library preparation kit. LABIM17 was sequenced using the MiSeq platform (Biotechnology Research and Innovation, Brazil) with the MiSeq reagent v2 micro kit (300 cycles; Illumina). The total number of paired-end reads from the sequencing process was 3,111,712, and fragment sizes varied from 35 to 151 bp. Reads were subjected to quality analysis with FastQC v0.11.9 (4). Reads with Phred scores of less than 30 and with one or more ambiguities were removed, and reads were trimmed using Trimmomatic v0.39 (5). The reads were filtered to generate contigs using *de novo* assembly methods with IDBA v1.1.3 (6). Assemblies with different k-mer sizes were compared using QUAST v5.0.2 (7), and the best one (with 362 contigs and an *N*_50_ value of 54,841 bp) was selected for scaffolding. The average sequencing coverage was estimated to be 78-fold. The scaffolding phase was carried out using CONTIGuator v2.7 (8), with B. brevis NBRC 100599 (GenBank accession number NC_012491) as a reference genome. Annotation was performed using the NCBI Prokaryotic Genome Annotation Pipeline (PGAP). The genome was estimated to be 5,950,202 bp, with a G+C content of 47.5% and 5,477 coding sequences, and the average nucleotide identity (9) with the closest strain, Brevibacillus brevis ATCC 35690 (GenBank accession number MXAR00000000), was 97.79%. Default parameters were used for all software. Twelve putative gene clusters responsible for secondary metabolite biosynthesis were identified using antiSMASH 6.0 (10), seven of which were related to known clusters from the MIBiG database (11). It was possible to highlight clusters linked to the synthesis of petrobactin, octapeptin, tyrocidine, gramicidin, zwittermicin, aurantinin, and pacidamycin.

### Data availability.

The whole-genome project was deposited in GenBank with Sequence Read Archive (SRA) accession number SRR17297603, BioProject accession number PRJNA780906, BioSample accession number SAMN23203606, and GenBank accession number CP087980. The version described in this paper is the first version, CP087980.1.
